# Presentation of Infectious Keratitis to ED during COVID-19 Lockdown

**DOI:** 10.1155/2021/5514055

**Published:** 2021-06-08

**Authors:** Barry Power, Aaron Donnelly, Conor Murphy, Timothy Fulcher, William Power

**Affiliations:** ^1^Royal Victoria Eye and Ear, Dublin, Ireland; ^2^Mater Misericordiae University Hospital, Dublin, Ireland

## Abstract

**Objectives:**

To compare presentation of infectious keratitis during COVID-19 lockdown with previous years, assess relative severity, and compare outcomes between COVID-19 and pre-COVID-19 era groups.

**Methods:**

Acute presentations of infectious keratitis during a strict government-mandated COVID-19 lockdown period were analysed retrospectively (March–May 2020). Data were compared with the same periods in 2018-2019. The clinical notes of patients undergoing corneal scrapes were reviewed, and data were collected on treatment, culture growth, surgical interventions, visual outcomes, admission rates, and risk factors.

**Results:**

There were 37% fewer presentations of infectious keratitis to the ED in 2020 (*N* = 29, 47, and 45, respectively). Risk factor profiles and microbial data were similar across all periods. Admission rates and use of fortified antibiotics were lower in 2020. COVID-19 era cases recovered less vision (LogMAR 0.26, 0.67, and 0.45, respectively; *p* = 0.04) and were more likely to require surgical intervention (10%, 4%, and 2%, respectively; OR 3.4 (CI 0.7–17.9, *p* = 0.1)).

**Conclusion:**

A concerning fall in presentations of infectious keratitis to ED during the pandemic lockdown was observed. Though societal behaviour changed during the lockdown, our data suggest it is unlikely that the incidence of infectious keratitis fell significantly. It is unclear how and where these patients were treated. We postulate that lower levels of visual recovery and higher rates of surgical intervention may have been caused by delays in accessing care. To minimise avoidable ocular morbidity as COVID-19 resurges, we must communicate clearly with patients and health professionals on how to access available emergency eye care services.

## 1. Introduction

The COVID-19 global pandemic led to major alterations in the way emergency eye care is accessed. Western Europe was the epicentre of the pandemic during the spring of 2020. During peak periods, strict government lockdowns in many countries limited travel. Healthcare activity was restricted to essential work only. As a result, the number of patients presenting for emergency eye care decreased [1–6], and a greater proportion of presentations were considered to be potentially sight-threatening [1]. Similar trends have been observed in serious surgical and medical presentations to the main ED [7–9].

In addition to reduced hospital attendances due to the cancellation of routine and nonurgent services, many patients chose to avoid the hospital. Fear of contracting COVID-19 within the hospital has been shown to be the primary concern of patients. For many patients, anxiety over contracting COVID-19 outweighed concern for complications of other serious ongoing medical conditions [10, 11].

The global ophthalmology community had to make rapid adjustments to the delivery of eye care. The Royal College of Ophthalmologists and the American Academy of Ophthalmology both produced guidelines on prioritising emergency cases that required assessment during COVID-19-related restrictions [12, 13]. Painful and sight-threatening conditions make up the majority of this list. Urgent corneal cases requiring immediate care include active keratitis of any sort, corneal ulcers, corneal perforation or melt, corneal graft rejection, and foreign bodies that necessitate removal [13].

Infectious keratitis is a sight-threatening and time-sensitive condition. Cases that receive appropriate treatment promptly have better outcomes [14, 15]. We hypothesised that the global pandemic and resultant government lockdowns may have led to a reduction in infectious keratitis presentations to the ED and a delay in diagnosis, potentially causing worse outcomes.

In this study, we aim to

1. Compare presentation of infectious keratitis during COVID-19 lockdown with previous years,
2. Assess relative severity and compare outcomes between COVID-19 and pre-COVID-19 era groups.

## 2. Methods

We compared the emergency department presentation of suspected infectious keratitis during COVID-19 pandemic with the two preceding years. We identified all patients presenting with suspected infectious keratitis requiring corneal scrapings during the months of March, April, and May—the local peak of the government-mandated lockdown. We compared them with patients who required corneal scrapings during the same periods in 2019 and 2018. Data were collected from the microbiology labs of the two major ophthalmic hospitals that serve a combined catchment area of 2 million. One centre is a standalone eye unit; the other is part of a large tertiary referral centre.

Both centres operated appointment-only emergency eye departments during the lockdown. Patients, GPs, and opticians were asked to e-mail or phone to access emergency care appointments. Appointments, if clinically indicated, were typically arranged within 24 hours. Triage was performed by ophthalmologists or clinical nurse specialists.

We recorded details of all emergency department corneal samples sent for culture and sensitivity (C+S) from microbiology lab records. Clinical notes for all cases were reviewed. The following clinical data were collected: date and location of presentation, presenting and final best-corrected visual acuity (BCVA), contact lens use, presence of risk factors, initial treatment, need for admission, additional surgical interventions (e.g., corneal glue and transplant), and microbial growth.

## 3. Results

Suspected infectious keratitis presentations, requiring corneal culture and sensitivity (C+S), were reduced by 37% in the lockdown group, compared with the average of the same period in preceding years. There was no difference in the proportion of infections associated with contact lens (CL) use or underlying risk factors, although there were fewer cases associated with trauma in 2020.  Table 1 summarises the data.

Patients presenting during the COVID-19 lockdown were less likely to be admitted after corneal scrapings were acquired (52%, 62%, and 69%, respectively). A lower proportion of patients were prescribed fortified antibiotics as first-line therapy (Table 2).

Patients presenting during the lockdown had a significant reduction in LogMAR visual recovery (LogMAR 0.26, 0.67, and 0.45, respectively; *p* = 0.04; Kruskal–Wallis). Ten percent of patients required emergency surgery (corneal glue, transplant of any form) in 2020, a higher rate than previous years. The odds ratio of requiring emergency surgery in 2020 compared with 2018-2019 was 3.4 (CI 0.7–17.9, *p* = 0.1) (Table 3).

Table 4 summarises the C + S results. There were a greater proportion of Gram-negative infections in 2020 compared with 2019 and 2018, although the overall rate of culture positivity was reduced during lockdown.

## 4. Discussion

We expected overall ED presentations to fall during the lockdown as patients with less acute issues stayed at home or were managed remotely. However, our data suggest that the lockdown also led to reduced presentations of acutely painful and debilitating conditions such as infectious keratitis.

There were 37% fewer presentations with suspected infectious keratitis during a three-month strict COVID-19 lockdown compared with the same period in the preceding two years. Significant societal behaviour changes occurred during the lockdown (working from home and reduction in socialising and sporting activities). We had hypothesised that CL use may fall during the lockdown. However, contact lens-related infection rates did not change between the periods suggesting that reduced contact lens use was not a major factor. The proportion of cases with other documented risk factors was also similar across the 3 time periods. A possible factor contributing to the observed reduction in cases was a lower rate of ocular trauma during the lockdown period. There were no cases of infectious keratitis secondary to trauma in the COVID-19 lockdown group compared with 2 (4%) in 2019 and 3 (7%) in 2018. This small difference is not enough to explain the reduced presentations alone.

A number of other studies have documented an overall reduction in presentations to eye emergency services [1–6]. Wickham et al. reported a relative increase in presentations of sight-threatening conditions—primarily due to a fall in less acute triage categories [1]. Interestingly, they also noted a 62% reduction in retinal detachments over 4 weeks, despite the temporary closure of some local vitreoretinal services. We agree with their assertion that lockdown behaviour change was unlikely to lead to such a significant fall in retinal detachment incidence. Posyer et al. reported a reduction in presentation of retinal tears combined with an increase in macula-off detachments, potentially indicating delayed presentations [4]. Hattenbach et al. reported a reduction in presentation of retinal detachment, acute glaucoma, central retinal artery occlusion, and anterior ischemic optic neuropathy [6]. All of these data suggest that some patients with debilitating and sight-threatening conditions did not access emergency eye care during the COVID-19 lockdown. Given the similar risk factor profiles of our patients, we did not detect a trend, which would support behaviour change leading to a reduced incidence.

This raises the question “did these patients receive treatment for their condition and if so where?” Robust phone triage pathways were in place in both hospitals. Patients reporting a red, painful eye with reduction in vision were offered an emergency appointment within 24 hours. During the lockdown, it is possible some patients sought advice from general practitioners over the phone. Chloromycetin is the most common first-line therapy prescribed in general practice and has good Gram-positive coverage. We detected lower overall growth rates and lower levels of Gram-positive infections in 2020. Treatment before presentation could help to explain this trend.

Unsurprisingly, there was a fall in ophthalmic admission rates in 2020. This decrease was expected, as both patients and clinicians were keen to minimise inpatient hospital stays during the peak pandemic period. The lower admission rate may explain the reduced use of fortified antibiotics (ceftazidime and vancomycin) as first-line therapy.

Our study suggests potentially poorer outcomes for infectious keratitis patients during the COVID-19 lockdown period. LogMAR visual improvement was significantly lower in 2020 compared with prior two periods. In addition, there was a greater likelihood of requiring surgical intervention in the lockdown period compared with the preceding years. It is possible that lockdown restrictions led to delays in presentation. In some cases, this may have resulted in more advanced disease requiring surgical intervention. It is also possible that visual recovery was inferior during lockdown due to deviations from the typical standard of care—fewer patients admitted and fewer patients treated with fortified antibiotics. Adherence to hourly and overnight drop regimes may not have been as strict with some patients treated as outpatients.

There are some limitations to note in the data. The visual data must be interpreted with caution, as the follow-up time was variable; thus, some final visual acuities may not have been accurate. Only patients with presenting and follow-up visual acuity were included in the visual data (7 patients excluded with incomplete visual data). The presence of some outliers in the visual results may also skew the data due to the relatively low numbers.

The first lockdown phase in Western Europe was unprecedented. The public and healthcare providers were, understandably, fearful of the potentially devastating impact of COVID-19. It is clear that this fear, combined with uncertainty over access to emergency care, led to a decrease in patients availing of emergency eye care. In this study, we demonstrate a fall in presentations of infectious keratitis, as well as significantly worse visual outcomes and a potentially increased likelihood of surgical intervention. Considering this, we must plan ahead for similar scenarios in the future. As many countries in Europe move into further lockdowns, communication with patients, GPs, optometrists, and other healthcare providers is critical.

Locally, we have contacted general practitioners to reiterate and reemphasise our altered emergency referral pathways. The message that EDs are open for all medical conditions has been emphasised by the Chief Medical Officer in multiple public addresses. The RCOphth has produced a timely “second wave” guidance document where they stress the importance of communicating with the public and other healthcare providers the importance of accessing ophthalmic care when indicated [16]. Follow-up audits of infectious keratitis rates could measure success in getting this message across to the public. We must emphasize the available services, provide clear information on how to access them, and encourage appropriate attendance to minimise unnecessary ocular morbidity.

## Figures and Tables

**Table 1:** Risk factors.

	2020	2019	2018
Total corneal C + S	29	47	45

Risk factor *N* (%)			
CL use	9 (31)	16 (34)	12 (28)
Corneal surface disease	2 (7)	2 (4)	4 (9)
Previous corneal surgery	3 (10)	3 (6)	2 (4)
RCES	1 (3)	0	0
Previous keratitis	1 (3)	1 (2)	0
Neurotrophic cornea	2 (7)	7 (15)	2 (4)
Trauma	0	2 (4)	3 (7)

C + S, culture and sensitivity; RCES, recurrent corneal erosion syndrome.

**Table 2:** Clinical details.

	2020	2019	2018
Admitted *N* (%)	15 (52)	29 (62)	29 (69)

Treatment *N* (%)			
Cef/Vanc	13 (45)	25 (53)	28 (64)
Ofloxacin	14 (48)	17 (36)	7 (16)
Brolene/PHMB	1 (3)	3 (6)	1 (2)
Anti viral	0	5 (11)	5 (11)
Anti fungal	1 (3)	2 (4)	1 (2)
Not recorded	0	0	3 (7)

Treatment, all patients receiving described treatment, including combination therapy. Cef/Vanc, ceftazidime/vancomycin; PHMB, polyhexamethylene biguanide; others, antifungal and antiviral therapy.

**Table 3:** Outcomes.

	2020	2019	2018
Mean LogMAR BCVA			
Presentation	1.04	1.16	1.15
Final	0.78	0.49	0.7
Change^*∗*^	0.26	0.67	0.45

Surgical intervention *N* (%)	3 (10)	2 (4)	1 (2)

BCVA, best-corrected visual acuity. ^*∗*^*P* value = 0.04.

**Table 4:** Microbiologic profiles of corneal cultures.

	2020	2019	2018
Cultures taken	29	47	45

Positive culture *N* (%)	9 (31%)	23 (49%)	25 (56%)

Gram-positive	3 (33)	12 (52)	13 (52)
*Streptococcus*	0	2 (9)	2 (8)
*S. aureus*	2 (22)	3 (13)	5 (20)
*S. epidermidis*	0	6 (26)	3 (12)
Others^a^	1 (11)	1 (4)	3 (12)

Gram-negative	5 (55)	9 (39)	10 (40)
* Pseudomonas*	0	1 (4)	1 (4)
* Moraxella*	5 (55)	6 (26)	6 (24)
Others^b^	0	2 (9)	1 (4)

Fungi	1 (11)	2 (9)	1 (4)
Yeast	1 (11)	0	1 (4)
Filamentous	0	1 (4)	0

Acanthamoeba	0	1 (4)	1 (4)

^a^Others, Gram-positive organisms included *Corynebacterium striatum*, Diphtheroids, *Propionibacterium*, and *Micrococcus luteus*. ^b^Others, Gram-negative organisms included *Stenotrophomonas* and *Serratia*.

## Data Availability

Data is available upon request.

## References

[1] Wickham L., Hay G., Hamilton R. (2020). The impact of COVID-19 policies on acute ophthalmology services-experiences from Moorfields Eye Hospital NHS foundation trust. *Eye*.

[2] Mehrotra A., Chernew M., Linetsky D. (2020). *The Impact of the COVID-19 Pandemic on Outpatient Visits: A Rebound Emerges*.

[3] Mehrotra A., Chernew M., Linetsky D. (2020). *The Impact of the COVID-19 Pandemic on Outpatient Visits: Practices Are Adapting to the New Normal*.

[4] Poyser A., Deol S. S., Osman L. (2020). Impact of COVID-19 pandemic and lockdown on retinal detachments. *Eye*.

[5] Pellegrini M., Roda M., Lupardi E., Di Geronimo N., Giannaccare G., Schiavi C. (2020). The impact of COVID‐19 pandemic on ophthalmological emergency department visits. *Acta Ophthalmologica*.

[6] Hattenbach L. O., Heinz P., Feltgen N. (2020). Auswirkungen der SARS-CoV-2-pandemie auf die ophthalmologische versorgung in Deutschland [impacts of the SARS-CoV-2 pandemic on ophthalmic care in Germany]. *Ophthalmologe*.

[7] Vanni G., Legramante J. M., Pellicciaro M. (2020). Effect of lockdown in surgical emergency accesses: experience of a COVID-19 hospital. *In Vivo*.

[8] Morelli N., Rota E., Terracciano C. (2020). The baffling case of ischemic stroke disappearance from the casualty department in the COVID-19 era. *European Neurology*.

[9] Tam C. F., Cheung K. S., Lam S. (2020). Impact of coronavirus disease 2019 (COVID-19) outbreak on ST-segment–elevation myocardial infarction care in Hong Kong, China. *Circulation: Cardiovascular Quality and Outcomes*.

[10] Wong L. E., Hawkins J. E., Langness S. (2020). Where are all the patients? addressing Covid‐19 fear to encourage sick patients to seek emergency care. *NEJM Catalyst*.

[11] Fung T. H. M., Kuet M. L., Patel M. K., Puri P. (2020). Addressing COVID‐19 fear to improve clinic attendance for patients with wet age‐related macular degeneration. *Acta Ophthalmologica*.

[12] Royal College of Ophthalmologists (2020). *Management of Ophthalmology Services During the COVID-19 Pandemic*.

[13] American Academy of Ophthalmology (2020). *New Recommendations for Urgent and Nonurgent Patient Care*.

[14] Miedziak A. I., Miller M. R., Rapuano C. J., Laibson P. R., Cohen E. J. (1999). Risk factors in microbial keratitis leading to penetrating keratoplasty. *Ophthalmology*.

[15] Vajpayee R. B., Dada T., Saxena R. (2000). Study of the first contact management profile of cases of infectious keratitis: a hospital-based study. *Cornea*.

[16] The Royal College of Ophthalmologists (2020). *Preparing Eye Care Services for a Potential COVID-19 Second Wave*.

